# Skeletal Muscle Lymphoma Presenting with Chronic Compartment Syndrome of Leg after Trauma

**DOI:** 10.1155/2018/4078672

**Published:** 2018-04-01

**Authors:** Jhong-You Li, Chung-Liang Li, Chun-Kuan Lu

**Affiliations:** Kaohsiung Medical University Hospital, No. 100 Tzyou 1st Road, Kaohsiung 807, Taiwan

## Abstract

Compartment syndrome may be acute or chronic based on the clinical course and etiology. Here, we report the first known case to be diagnosed with skeletal muscle-derived B-cell lymphoma presenting with chronic compartment syndrome after trauma. A 62-year-old woman sought medical attention due to a one-month history of painful left lower leg swelling and paresthesia of the medial side of the foot after falling over. The patient underwent fasciotomy and debridement under the preoperative diagnosis of fasciitis and myositis with associated compressive neuropathy. Preoperative laboratory tests were within normal limits. Postoperative pathologic examination and bone marrow aspiration revealed B-cell lymphoma with bone marrow involvement postoperatively. Tumor lysis syndrome took place, presenting with drowsiness, poor appetite, and oliguria, after the operation along with multiple organ failure. Awareness of the differential diagnoses of compartment syndrome in such clinical situation is crucial because it may lead to different examination and treatment plan preoperatively.

## 1. Introduction

Compartment syndrome of the leg is a complication caused by fracture, ischemic reperfusion injury, soft tissue trauma, external compression, burn, or even bleeding disorders [1]. The diagnosis is mainly based on history, clinical examination, and occasionally intracompartment pressure (ICP) measurement. Delay in treatment of compartment syndrome may lead to muscle necrosis, limbs amputation, and even worse, rhabdomyolysis, renal failure, and death [2].

Extranodal non-Hodgkin lymphoma (NHL) is well reported with sites involved including the central nervous system, gastrointestinal tract, lung, skin, or bone [3]. Skeletal muscle-derived extranodal NHL is extremely rare [4], and few of the reported cases in the literature present with compartment syndrome. Correct diagnosis may lead to different evaluation and treatment plans.

## 2. Case Presentation

A 62-year-old woman with underlying disease of hypertension presented with a one-month history of painful left lower leg swelling and paresthesia of the medial side of the foot after falling over. There were no associated B symptoms noted (known as fever, weight loss, or night sweats). Physical examination disclosed an ill-defined, tense, and tender swelling of left calf with local heat. Dorsalis pedis artery and posterior tibial artery pulsations were intact, but there was numbness over the medial sole and medial 3 toes.

**Figure 1:**
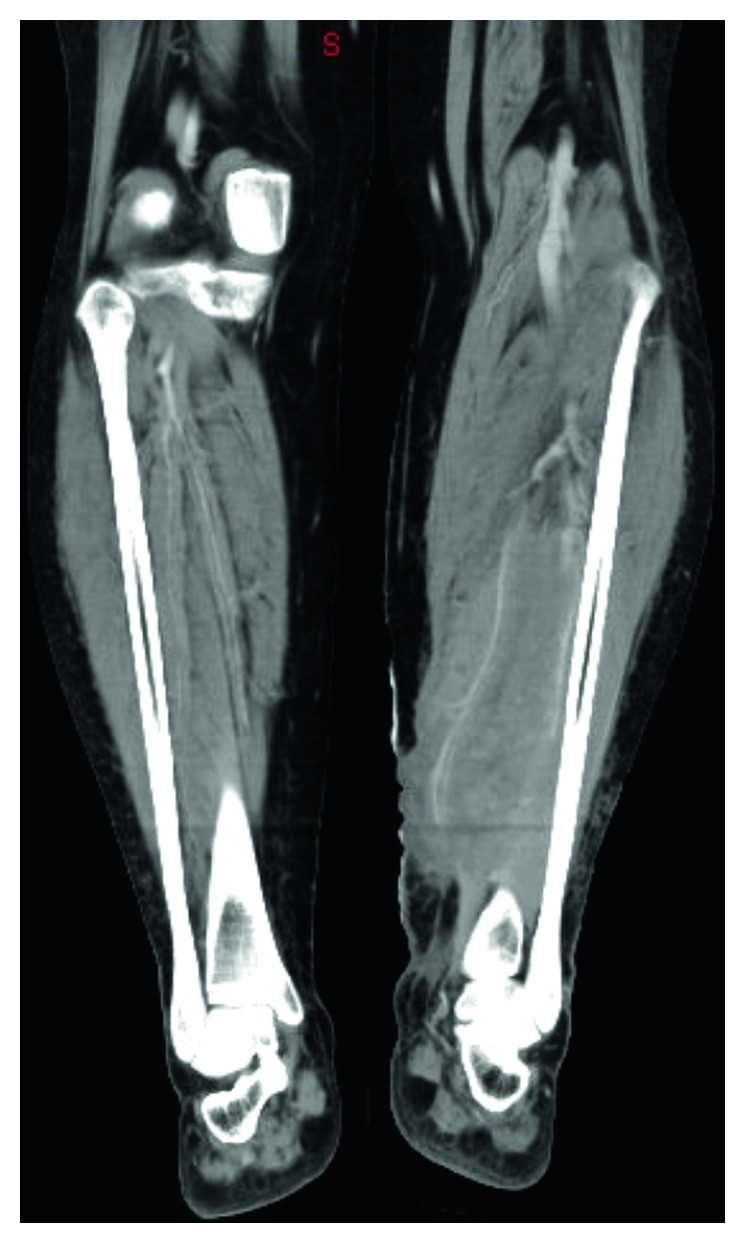
Contrast-enhanced CT of lower extremities. CT shows hypodense lesions and fluid collection in the medial aspect of the posterior compartment of the left lower leg.

The patient was admitted for diagnostic workup, including laboratory examination and contrast-enhanced computed tomography (CT) of the lower extremities. Preoperative liver, renal function, and white blood cell (WBC) count were within normal limits without evidence of blast or atypical lymphocyte. Contrast-enhanced CT showed hypodense lesions and fluid collection in the medial posterior compartment of the left lower leg (Figure 1). Under the tentative diagnosis of fasciitis and myositis with associated compressive neuropathy, scheduled operation with debridement and fasciotomy were performed.

**Figure 2:**
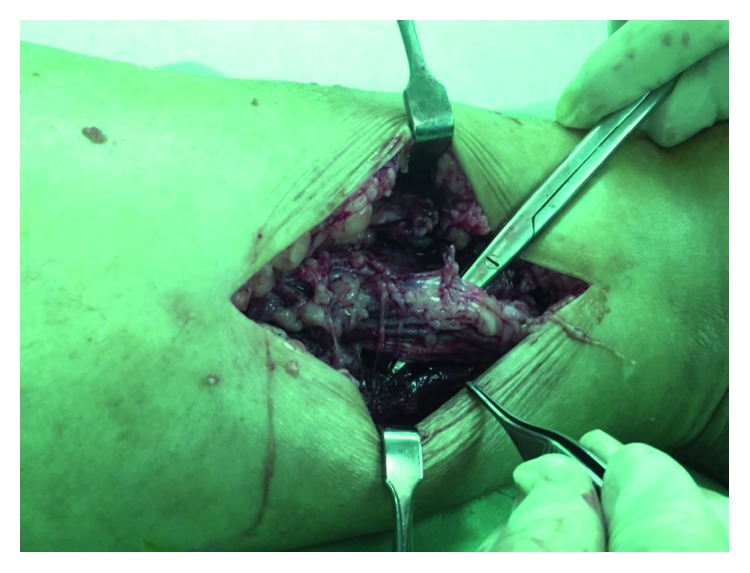
Myonecrosis of the left lower leg. Intraoperative finding demonstrated myonecrosis of the deep posterior compartment and the posterior tibal nerve surrounded by necrotizing tissue (status after posterior tibial nerve neurolysis).

During operation, subcutaneous fatty necrosis with induration was noted and evacuated. Upon dissection into the deep compartment of posterior leg, myonecrosis of nearly the entire compartment was discovered, and the tibial nerve was surrounded by the necrotic soft tissue (Figure 2). Fasciotomy and thorough debridement were performed. Microbiology and pathologic studies were done. Skin was approximated, and a Jackson-Pratt drain was inserted into the posterior compartment in order to monitor wound condition and prevent further hematoma formation.

However, drowsiness, poor appetite, and oliguria developed 2 days after the operation while the patient remained afebrile. Repeated laboratory workup revealed leukocytosis with atypical lymphocyte. Abnormal liver function tests and acute renal failure postoperatively suggested the possibility of rhabdomyolysis, severe sepsis, or even tumor lysis syndrome. Additional laboratory studies showed electrolyte imbalance with hypocalcemia, hyperphosphatemia, hyperuricemia, and markedly elevated lactate dehydrogenase (LDH) (19,047 U/L) without significant elevated creatine phosphokinase (CPK) level. Microbiologic examinations were all negative.

**Figure 3:**
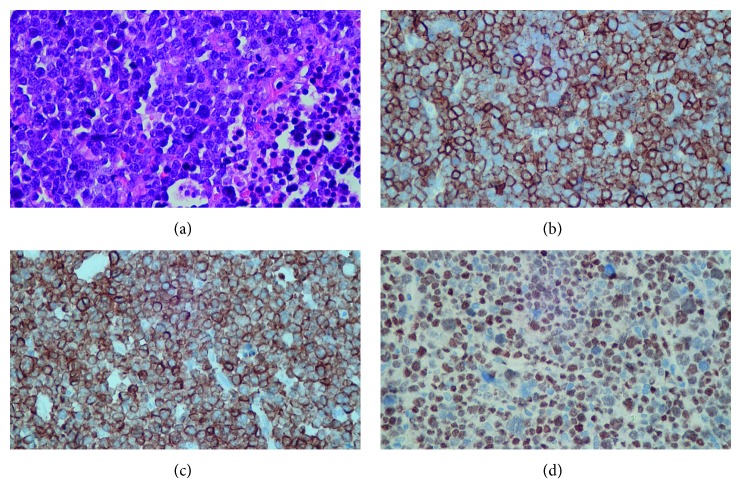
Microscopic view of intraoperative soft tissue specimen. (a) Infiltration of neoplastic cells in the soft tissue, characterized by high nuclear-to-cytoplasmic ratio in hematoxylin and eosin (H&E) stain. Positive in immunochemical stain: CD20 (b), bcl-2 (c), and c-myc (d) in immunochemical stain.

**Figure 4:**
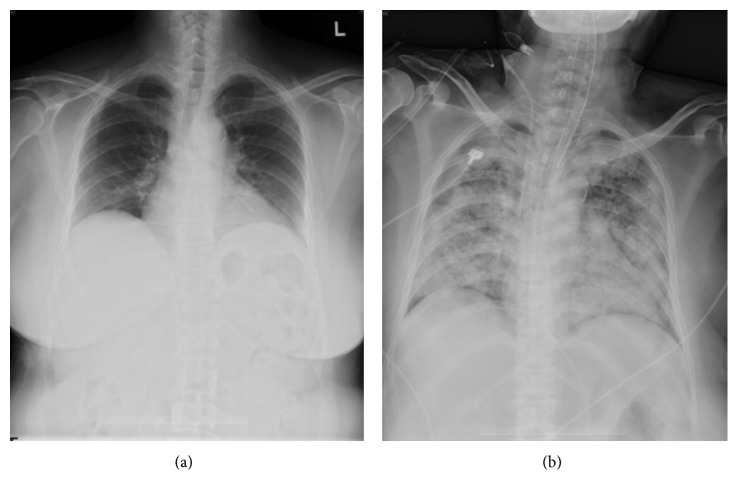
Chest radiograph revealed ARDS lung. (a) preoperative; (b) postoperative.

Pathologic examination of the deep posterior compartment of the lower leg confirmed the diagnosis of B-cell lymphoma with features intermediate between diffuse large B-cell lymphoma and Burkitt lymphoma (Figure 3). Hematologist and nephrologist were immediately consulted for suspected B-cell lymphoma with tumor lysis syndrome. Bone marrow aspiration on the next day showed abundant blast-like cells (56%) in the smear. Chest CT was arranged for further evaluation, and it revealed probably lymphadenopathy at the right submental area and pretracheal region. Along with the peripheral blood smear and pathologic report, acute lymphoblastic leukemia or lymphoma with secondary muscle involvement was confirmed. Emergent hemodialysis was performed on hospital day 12 due to tumor lysis syndrome with associated electrolyte imbalance. The patient's family members refused chemotherapy. On hospital day 22, the patient died because of intractable gastrointestinal bleeding and multiple organ failure in the intensive care unit (Figure 4).

## 3. Patient Declaration Statement

The authors certify that they have obtained all appropriate patient consent forms. In the form, the patient has given her consent for her images and other clinical information to be reported in the journal. The patients understand that their names and initials will not be published and due efforts will be made to conceal their identity, but anonymity cannot be guaranteed.

## 4. Discussion

To date, only few cases of lymphoma originating in the skeletal muscle presenting with compartment syndrome have been reported. During our literature review, 7 cases of compartment syndrome caused by hematologic diseases in the skeletal muscle were found [3–7]. Only two of theses cases had lymphoma and the others had leukemia (Including both acute lymphoblastic leukemia (ALL) and acute myeloid leukemia (AML)). Over half of the patients were either children or teenagers (aged 9, 11, 11, and 20 years). The most commonly involved sites were the forearm (three cases) and legs (two cases). A case with buttock and thigh involvement was also reported [8]. To the best of our knowledge, this is the first case of skeletal muscle-derived B-cell lymphoma that presents with compartment syndrome after trauma.

Compartment syndrome may be acute or chronic based on the clinical course and etiology. Acute compartment syndrome is a limb-threatening and potentially life-threatening condition caused by fracture, ischemic reperfusion injury, soft tissue trauma, external compression, burn, or even bleeding disorders [1, 2]. Chronic compartment syndrome is a relative medical nonemergency and mostly common in athletes with repetitive motions [9]. The characteristics of chronic compartment syndrome are pain, cramping, or even numbness with exercise that are relieved with rest. The anterior and deep posterior compartments of lower extremities are the most frequently affected sites, and fracture or other types of trauma are the precipitating factors [9].

We propose an idea that apart from repetitive exercise, chronically increased intracompartment pressure may also induce symptoms and signs of chronic compartment syndrome in this case. Chronic compartment syndrome gradually developed due to chronic bleeding and poor healing secondary to lymphoma infiltration after the trauma. Additional studies such as muscle biopsy, CT, or magnetic resonance imaging (MRI) should be considered if the disease course is prolonged or disproportionate lesion. Although surgical debridement and fasciotomy is widely recommended in the presence of compartment syndrome [1–3, 5–9], muscle biopsy for pathology proof is crucial before or during the operation. Definite treatment of lymphoma/leukemia usually required systemic chemotherapy that may be contraindicated in the acute setting of compartment syndrome as it might worsen the tissue swelling [6]. Moreover, there is a risk of tumor lysis syndrome whether chemotherapy or surgery is instituted. Close monitoring of urine output and renal function along the treatment course is needed.

## 5. Conclusion

Primary skeletal muscle lymphoma is extremely rare, accounting for approximately 1.5% of NHLs. And they usually have large cell histology features and have been associated with a poor prognosis [10]. We have reported a posttraumatic compartment syndrome secondary to primary muscle lymphoma. The case highlights the possibility of malignancy-related compartment syndrome even after a specific trauma history.
